# Reversible alopecia in Vogt-Koyanagi-Harada disease and sympathetic ophthalmia

**DOI:** 10.1186/1869-5760-3-41

**Published:** 2013-03-15

**Authors:** Chiu-Tung Chuang, Po-Sian Huang, Shih-Chou Chen, Shwu-Jiuan Sheu

**Affiliations:** 1 Department of Ophthalmology, Kaohsiung Veterans General Hospital, 386, Ta-Chung 1st Road, Kaohsiung, 813, Taiwan; 2 School of Medicine, National Yang-Ming University, Taipei, 112, Taiwan

## Abstract

**Background:**

Vogt-Koyanagi-Harada (VKH) disease and sympathetic ophthalmia (SO) are both autoimmune disorders targeting melanin-bearing cells, even though their etiologies are different. Both shared many ocular and systemic manifestations, including integumentary findings. Most of the literature focused on the ocular manifestations and related treatment. Alopecia was seldom mentioned.

**Findings:**

We report one case of VKH disease and one case of SO. Both developed severe alopecia and early sunset glow fundus, which are probably due to incomplete treatment. Fortunately, the alopecia improved soon after systemic steroid treatment.

**Conclusions:**

Early and complete treatments are important in the management of VKH or SO and prevent integumentary manifestation. Alopecia can be reversible after steroid treatment in time.

## Findings

### Introduction

Vogt-Koyanagi-Harada (VKH) disease and sympathetic ophthalmia (SO) are autoimmune disorders, targeting melanin-bearing cells. Both diseases are characterized by immunologic dysregulation [1]. The reports from literature suggest that the disorders have different etiologies with similar ocular and systemic manifestations [1-3]. According to the diagnostic criteria, patients with VKH should have no history of surgery or trauma. In addition, neurological and integumentary findings are the necessary condition to fit the complete form of VKH. Ocular depigmentation was defined as the late manifestation of VKH cases [4]. Ocular depigmentation and integumentary findings (alopecia, poliosis, and vitiligo) are supposed to be the sequelae of destruction of melanin-bearing cells by the autoimmune attack. Contrary to VKH, alopecia was seldom mentioned in case of SO [3,5,6]. Based on its pathogenesis, it is reasonable to expect integumentary manifestations in SO case. Here we report two interesting cases presenting reversible alopecia in a VKH patient and an SO patient.

### Case 1

**Figure 1:**
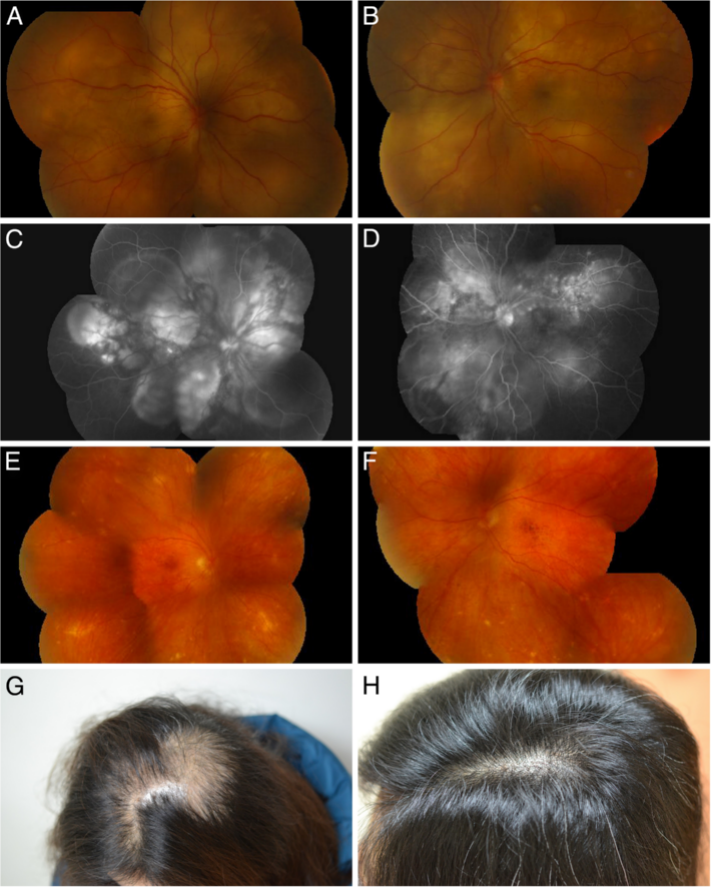
**Clinical manifestations of the patient with VKH.** (**A,B**) Initial fundus examination showed multiple bullous retinal detachment in both eyes. (**C,D**) Fundus fluorescent angiography showed multiple pinpoint leakage and late dye pooling in both eyes. (**E,F**) Remarkable sunset glow fundus in both eyes. (**G**) Severe alopecia developed 5 months after onset. (**H**) Alopecia improved after systemic steroid.

A 45-year-old female visited our clinic on July 10, 2011 with the chief complaint of acute onset of blurred vision in both eyes for 1 week. There was no other related symptom except tinnitus. She had history of drug abuse and radial keratotomy for myopia 20 years ago (−2D before operation). Otherwise, she was quite well systemically. On presentation, her vision was 6/60 (6 + 4.75, −1.75 × 175) in the right eye and 6/60 (+4.25, −2.5 × 170) in the left eye. Ophthalmic examination showed mild anterior chamber reaction and multiple lobules of serous retinal detachments in both eyes (Figure 1A,B). Fluorescent angiography (FAG) showed multiple pinpoint hyperfluorescent spots with severe leakage and pooling at late phase in both eyes (Figure 1C,D). Vogt-Koyanagi-Harada disease was the impression based on the clinical pictures and characteristic FAG findings. Admission was scheduled on July 11 due to personal reason. Unfortunately, she called at ER for fever (38°C), chills, severe headache, and neck rigidity on July 10. Lumbar puncture showed pleocytosis (WBC count, 390 mm^3^; RBC count, 20 mm^3^; Pandy’s test (+); protein, 139; sugar, 70). No pigment-laden macrophage was found in the cytological study. Brain MRI was negative for space-occupying lesions or inflammation. Systemic workup including general physical examination, complete blood counting, differential classification, blood biochemical tests, rheumatic factor, anti-nuclear antibody, human immunodeficiency virus, venereal disease research laboratory test, herpes virus immunoglobulin, and chest radiography were within normal ranges. The initial report of tuberculosis (TB) polymerase chain reaction (PCR) from aqueous fluid was negative. Systemic intravenous methylprednisolone pulse (MTP) therapy was given 250 mg every 6 h for 3 days. The meningismus and serous detachment resolved after pulse (MTP) therapy and the vision improved a lot. Repeated cerebrospinal fluid (CSF) analysis on July 14 showed decreased pleocytosis (WBC count, 241 mm^3^; RBC count, 1 mm^3^, Pandy’s test (−); sugar, 101). Just after completing MTP therapy, the official report of TB PCR from the outside laboratory announced positive result. Infection specialist was consulted, who repeated the TB PCR on CSF. Although the results of tuberculin test, PCR, and culture on CSF were all negative for TB, the internist decided to give her four combined anti-tuberculosis medication for 6 months. Besides, oral and topical prednisolone was tapered gradually. However, the patient did not comply well with the treatment due to the side effects of steroid and allergic reaction to ethambutol. Progressive hair loss was noted 5 months later (Figure 1G). At this time, the vision was 6/10 (−1.25, −1.5 × 75) in the right eye and 6/12 (−2.5, −1.0 × 70) in the left eye. There was persistent anterior uveitis with secondary glaucoma. The fundus showed sunset glow appearance with multiple small atrophic lesions (Figure 1E,F). The patient agreed to receive and comply with large dose of systemic steroid (1 mg/kg) again. The alopecia improved soon after repeated systemic steroid (Figure 1H), but recurrent anterior uveitis persisted and remarkable sunset glow fundus developed in both eyes. Intravitreal injection of Ozurdex (dexamethasone implant 700 μg, Allergan Inc., Irvine, USA) was given in both eyes to replace the systemic steroid as the patient lost compliance again ever since her alopecia improved. The anterior uveitis was controlled after dexamethasone (Ozurdex) implant in both eyes.

### Case 2

**Figure 2:**
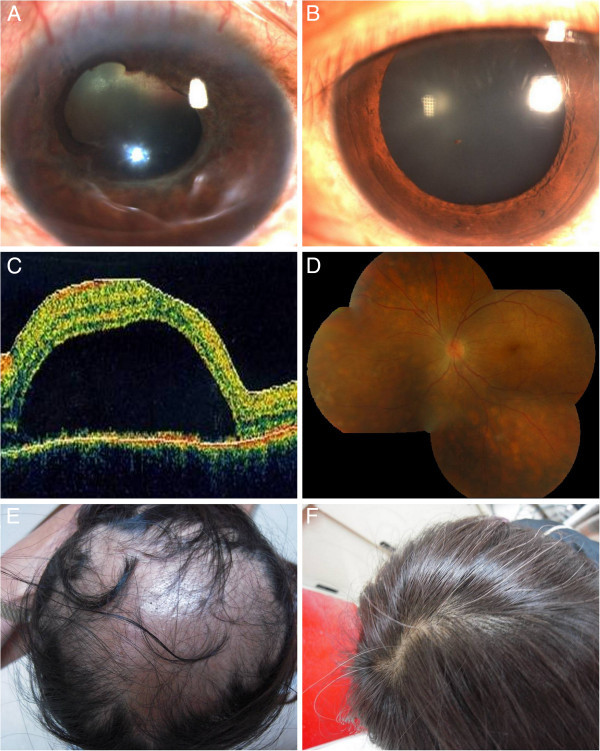
**Clinical manifestations of the patient with sympathetic ophthalmia.** (**A**) Atrophic change of right eye with posterior synechiae, lens opacity, and vitreous hemorrhage. (**B**) Slit lamp biomicroscopic examination showed keratic precipitate, trace cell in the anterior chamber in the left eye. (**C**) Optical computerized tomography of left eye showed serous retinal detachment. (**D**) Fundus examination showed remarkable sunset glow fundus with multiple atrophic lesions. (**E**) Severe alopecia developed 2 months after trauma in the right eye. (**F**) Alopecia improved after systemic steroid treatment.

A 41-year-old female visited our emergency room for blurred vision in her right eye after blunt injury inflicted by her boyfriend two days ago. She has past history of hyperthyroidism after treatment. At presentation, there was light perception only in the right eye and 20/30 (naked eye) in her left eye. Ophthalmic examination showed severe subconjunctival hemorrhage and hyphema with exudates in the anterior chamber of right eye. The fundus was invisible due to dense vitreous hemorrhage. Her left eye was unremarkable. Orbital CT showed irregular posterior contour of the right eye and surgical exploration was suggested under the impression of highly suspected eye ball rupture. Unfortunately, she refused and lost to follow up until 1 month later when she came back for blurred vision in her left eye. Ophthalmic examination showed no light perception and bulbar atrophy in the right eye, 6/10 with keratic precipitate, trace cell in the anterior chamber, and disk swelling in the left eye (Figure 2A,B). MTP pulse therapy was suggested based on the diagnosis of sympathetic ophthalmia. The patient refused admission and received topical and oral prednisolone (15 mg TID) instead, then lost to follow up for another 2 weeks when the vision deteriorated to 6/60 with serous retinal detachment (Figure 2C). The patient did not show up for scheduled MTP treatment again. She came back 1 month later with sunset glow fundus in the left eye and severe alopecia (Figure 2D,E). According to the patient, she never received oral steroid treatment by the dermatologist due to alopecia before this visit. The alopecia resolved after intensive oral steroid treatment (Figure 2F). The best corrected vision was no light perception in the right eye and 6/10 in the left eye 5 months after trauma. There were multiple recurrences of anterior uveitis with remarkable fundus depigmentation in her left eye.

### Discussion

Early and complete treatment is the key in the management of both VKH and SO diseases. Even with the development of new immunomodulatory agents, systemic steroid is the mainstay of treatment [7]. Lai reported that the duration of at least 6 months is a key factor in reducing chances of recurrence [8]. Other series reported that submaximal doses of inflammation suppressive therapy are sufficient to suppress clinically apparent disease but not the underlying lesion process. This explains the propensity for sunset glow fundus in a seemingly controlled disease [9].

Although integumentary manifestation is one of the major diagnostic criteria in VKH disease, the incidence of alopecia or poliosis is much less than the ocular manifestations [5,10-13]. In our previous reports, the incidence of integumentary manifestation was 15.4%, but only 3.2% in those whose who were treated within 1 month [11,14]. It is supposed to be the results of depigmentation of melanin-bearing cells as it is seen in the sunset glow fundus. As both VKH and SO are speculated to have an underlying T cell-mediated autoimmunity to uveal/retinal antigen, the melanin-bearing hair follicles may be targeted if the inflammation was not controlled well enough. Immediate immunosuppression by intensive steroid therapy could suppress the inflammation and prevent the total destruction of the hair cells. The presentation might be also influenced by the completeness of treatment [15]. The increased awareness of diagnosis and treatment of VKH might explain the rare case of severe alopecia. The consequent change of alopecia or poliosis had seldom been mentioned in the literature. There was one report about partially reversible sensorineural hearing loss in VKH disease from Ondrey et al. [16]. Our cases were obviously under-treated before the development of alopecia. Fortunately, the alopecia resolved after systemic steroid therapy. Their experiences can be used as one of the examples to persuade patients with VKH and SO to keep on complete treatment.

### Conclusion

Early and complete treatments are important in the management of VKH or SO and prevent integumentary manifestation. Alopecia can be reversible after steroid treatment in time.

### Consent

Written informed consent was obtained from the patient for publication of this report and any accompanying images.

## Competing interest

The authors do not have any financial conflict or interest in the subject matter in the manuscript, and do not have any commercial or propriety interest in the product or company.

## Authors’ contributions

CT, PS, SC, and SJ are involved in the case collection. CT and SJ are responsible for the case design and analysis of data. SJ is also responsible for drafting the manuscript. All authors read and approved the final manuscript.
